# Optimal control methods for drug delivery in cancerous tumour by anti‐angiogenic therapy and chemotherapy

**DOI:** 10.1049/syb2.12010

**Published:** 2021-01-25

**Authors:** Pariya Khalili, Sareh Zolatash, Ramin Vatankhah, Sajjad Taghvaei

**Affiliations:** ^1^ School of Mechanical Engineering Shiraz University Shiraz Iran

## Abstract

There are numerous mathematical models simulating the behaviour of cancer by considering variety of states in different treatment strategies, such as chemotherapy. Among the models, one is developed which is able to consider the blood vessel‐production (angiogenesis) in the vicinity of the tumour and the effect of anti‐angiogenic therapy. In the mentioned‐model, normal cells, cancer cells, endothelial cells, chemotherapy and anti‐angiogenic agents are taking into account as state variables, and the rate of injection of the last two are considered as control inputs. Since controlling the cancerous tumour growth is a challenging matter for patient's life, the time schedule design of drug injection is very significant. Two optimal control strategies, an open‐loop (calculus of variations) and a closed‐loop (state‐dependent Riccati equation), are applied on the system in order to find an optimal time scheduling for each drug injection. By defining a proper cost function, an optimal control signal is designed for each one. Both obtained control inputs have reasonable answers, and the system is controlled eventually, but by comparing them, it is concluded that both methods have their own benefits which will be discussed in details in the conclusion section.

## 1 INTRODUCTION

Cancer is the name of a group of diseases in which a fact causes an uncontrolled proliferation of cells that could invade other tissues of the body. There are over hundreds of different types of cancer, which is one of the leading causes of death around the world. In general, researchers are interested in the numerous fields of cancer control such as early diagnosis, control signals during treatment sessions, and possible final care. [1]. There are several ways to treat this disease such as open surgery, radiotherapy, immunotherapy and chemotherapy. Among these various ways of cancer treatment, chemotherapy that is related to the injection of certain medications called chemotherapy medicines is assessed as an efficient and inclusive method, and many researchers have investigated the effects of this medicines on the cancer dynamics. In general, the papers that present a new mathematical model with the subject of cancer dynamics behaviour analysis follow below procedure: First, some state variables are taken into account as the basic states that have great effects on the cancer dynamics. Second, the corresponding mathematical model is dynamically analysed via stability theorems around the points of equilibrium. As expected, it is concluded that the system tends to the death state without any treatments and to healthy state with drug injection in some cases. Third, the proposed system is compared in two situations of with and without drug injections, in order to prove the effects of drugs and whether all the states behave logically or not. In each mathematical model, various states are considered such as cells in the patient's body [immune cells, normal cells (NCs) and etc.], effective drugs, and of course, tumour cells. In the mathematical models proposed in this field, various states are considered. Each model is capable of justifying a particular manner of this illness.

Before reviewing recent mathematical models, let us discuss the free‐model control method, a common method for complicated system dynamics, such as cancer. Machine learning (ML) and artificial intelligence (AI) methods are approaches that are based only on the laboratory data and learning algorithms for classifications or detection [2, 3, 4]. Although these methods determine the closed‐loop solution based on the previous information, it does not provide any data on changes of other variables in patient's body during treatment. For this reason, differential equations, based on time variation, are presented to simulate such action, considering different states and variety of cancer effects in body, which will be discussed briefly in the following.

Models that simulate cancer behaviour are constantly being updated. The ones that choose chemotherapy as the main treatment have started with proposing a first‐order differential equation which are log kill [5] and *E*_max_ [6]; Norton & Simon [7] continued with considering variety of states along with different effects, such as metastasis [8], angiogenesis [9], fat [10], and delay differential equations [11]. The mathematical models can be helpful to find the best rate of drug injection during the patient's treatment for different purposes, like decreasing the toxicity risk of chemotherapy drugs [12] or finding the optimum drug rate injection. Therefore, it is a great challenge for researchers to obtain an accurate mathematical model and eventually controlling it. Among numerous models investigated in the field of simulating chemotherapy, some of them together with the procedures proposed to control the system are reviewed in the following.

One of the interesting field is finding the optimum treatment protocol for these patients. Various models are considered to extract the effective dose rate. The effect of immune cells, NCs and cancer cells (CCs) along with chemotherapy is mainly analysed in Reference [13] and controlled with basic optimal control approach by means of calculus of variations. In recent years, the effect of obesity as an effective and a significant factor in the treatment of cancer was also added to the previous model and mathematically analysed [10]. Afterwards, the basic optimal control approach (using calculus of variation method) was applied by defining a proper cost function, which was reducing the amount of CCs in the final day of treatment [14]. This optimum trajectory was later taking into account as the desired path for a sliding surface, and a robust sliding mode controller was designed to overcome the uncertainties and disturbances of the system [15].

The study of the three different control approaches for the three first‐order basic models was investigated in Reference [16]. Another strategy of using optimal approach in the treatment of cancer is to first apply a linear or nonlinear controller and then optimise the parameters by algorithms such as Genetic Algorithms (GA) [17]. It is worth‐noting that GA could also be the only tool for finding the best therapeutic protocol [18]. Recently, the combination of GA with other estimation algorithms for diagnosis [19] and adaptive approaches for prognosis [20] have been also an interesting field for researchers. Particle swarm optimisation for detection and fuzzy logic for prediction of cancer in patients are also utilised, respectively, in References [21,22].

The basic optimal control approach based on calculus of variations achieves the open‐loop solution for the controller design. However, some optimal control approaches such as state‐dependent Riccati Equation (SDRE) have a closed‐loop control structure and is suitable for complex nonlinear systems in which the controller design is accomplished in the presence of unmodeled dynamics. Since cancer mathematical models include highly nonlinear terms, and uncertainties and disturbances have significant effects in the system behaviour, SDRE controller seems to be a powerful tool. Itik et al. [23] obtained the control signal using SDRE on the dynamics as proposed in Reference [13] and analyzed the effect of weighting matrices in the cost function in order to find the solution which leads to the less drug injection and more CCs' reduction comparing to others.

The formation of new blood vessels is a natural process for the growth and healing, called angiogenesis. It is found that tumours induce the sprouting of new blood vessels in surrounding in order to supply tumour with oxygen and nutrients. Anti‐angiogenic refers to the drug that prevents the delivery of the signals from the tumour to the blood vessel, and diminishes the proliferation of new blood vessels, but this drug should not eliminate blood vessels because there must be a way to deliver the chemotherapy drug to tumour as a matter of destruction [24]. In fact, use of both anti‐angiogenic and chemotherapy drugs can cure cancer more effectively [25].

There are different strategies investigating the concurrent cooperation of chemotherapy and anti‐angiogenic experimentally or analytically. Like previous mentioned researches, some considered several states and proposed a mathematical model in the form of ODEs like, [26], and then designed optimal controllers for the obtained theoretical model, and some like, [27], succeeded in presenting a model in the field of cooperation of chemotherapy and anti‐angiogenic. This model was improved in [28]. One of the most comprehensive mathematical models for cancer dynamics with simultaneous impression of these two drugs was presented in Reference [29] by Pinho and co‐workers. In this model, the effects of chemotherapy and anti‐angiogenic drugs on NCs, CCs and endothelial cells (ECs) are considered, and a set of ODEs with five state variables is presented and mathematically analysed in detail. The stability and the equilibrium points of the proposed model are completely investigated in each subsystem. Afterwards, the idea of personalising the drug injection for each patient according to the individual optimum trajectory was investigated in Ref. [30] by means of adaptive controller design based on Pinho's model.

In this study, the five‐state mathematical model for cancer dynamics with the effects of chemotherapy and anti‐angiogenic injections is considered [29]. To attain the goal of reducing CCs, control signals are designed such that they will be optimum and have an acceptable consequence on other states. To do so, two optimal approaches are chosen: basic optimal control approach based on calculus of variations as an open‐loop [32] and SDRE as a closed‐loop controller. In the first control approach, steepest descent method is selected to calculate the solution in calculus of variations. The results of each are compared together.

This study is organized as follows: The mathematical model of cancer dynamics is introduced in Section 2. The basis of the two selected optimal control strategies is reviewed, and accordingly, the suitable control signals are designed in Section 3. In Section 4, simulation results of each proposed controller are figured and compared. Finally, Section 5 concludes the paper.

## 2 MATHEMATICAL MODEL

In this section, the mathematical dynamic model considered in this study, [29], is reviewed in the state‐space form. It should be mentioned that *x*_1_, *x*_2_ and *x*_3_ represent NCs, CCs and ECs, and express chemotherapy agent (CA) and anti‐angiogenic agent (AA), respectively. The non‐dimensional state‐space mathematical model of cancer includes five ODEs as follows:

(1) $$\begin{array}{l} {\dot{x}}_{1}=\alpha_{1}x_{1}\left(1-x_{1}\right)-q_{1}x_{1}x_{2}-p_{1}\left(x_{3},w\right)\frac{x_{1}y}{a_{1}+x_{1}}\\ {\dot{x}}_{2}=\alpha_{2}x_{2}\left(1-\frac{x_{2}}{1+\gamma x_{3}}\right)-q_{2}x_{1}x_{2}-p_{2}\left(x_{3},w\right)\frac{x_{2}y}{a_{2}+x_{2}}\\ {\dot{x}}_{3}=\beta x_{2}+\alpha_{3}x_{3}\left(1-x_{3}\right)-\frac{p_{3}x_{3}w}{a_{3}+x_{3}}\\ \dot{y}=\delta -\left(\xi +d_{1}\frac{x_{1}}{a_{1}+x_{1}}+d_{2}\frac{x_{2}}{a_{2}+x_{2}}\right)y\\ \dot{w}=\phi -\left(\eta +d_{3}\frac{x_{3}}{a_{3}+x_{3}}\right)w \end{array}$$

where *p*_*i*_(*x*_3_,*w*) = *p*_*i*0_+*p*_*i*1_ *x*_3_+*p*_*i*2_ *w*, *i* = 1,2.

It is assumed that NCs and CCs exhibit logistic proliferation with different rates ($\alpha_{1}$ and $\alpha_{2}$). The term $1+\gamma x_{3}$ in the second equation shows that the increase in ECs production enhances the tumour growth. The *q*_1_ and *q*_2_ represent the competitive rates, and −*x*_1_ *x*_2_ is the term that shows the competence between NCs and CCs. These two cells also get destroyed by CA with the rate of *p*_*i*_. There is a saturation term *x*_*i*_/(*a*_*i*_ + *x*_*i*_) in the end of the first two equations, where *a*_*i*_ is each saturation rate. The intention of saturation terms is that as $x_{i}\to \infty$, the fraction tends to value one. Note that *p*_*ij*_ (*i* = 1,2) is the contribution effect of killing *x*_*i*_by CA in the absence of *x*_3_ and *w*(*j* = 0), CA per concentration of *x*_3_ and *w*(*j* = 1,2).

ECs has a logistic growth (*x*_3_(1−*x*_3_)) with the rate of $\alpha_{3}$, and also duplicates based on tumour size ($\beta x_{2}$). Since ECs proliferation is less than NCs and CCs, $\alpha_{3}$ is assumed to be smaller than $\alpha_{1}$ or $\alpha_{2}$. Similar to NCs and CCs, ECs is killed with a saturation term in which AA is the matter of destruction (*x*_3_/(*a*_3_+*x*_3_)) with the rate of *p*_3_.

δ and ϕ are CA and AA rate of injections or control signals of the system. Both are destroyed depending on their half‐lives (ξ and η). CA also disappears by the effect of NCs and CCs with *d*_1_ and *d*_2_ rate, respectively. However, AA is lost only in ECs based on the model assumptions. Note that all the destruction terms in the last two equations appear as a saturation fraction along with their rates (*x*_*i*_/(*a*_*i*_ + *x*_*i*_), *i* = 1,2,3).

This model is able to justify the system's behaviour in the following categories confirmed by empirical data. Since the mathematical proofs are presented in Reference [29], the results are just listed in the following.

a) All states remain in the positive region with any nonnegative initial conditions.
b) Without any treatment courses, patient cannot be cured by immune body system and always leads to death undoubtedly.
c) AA is not able to cure cancer disease [31].
d) CA is capable of slowing down the progression of cancer under some circumstances.
e) CA with the aid of AA is a more powerful approach comparing to case (d) in cancer's remedy.

## 3 OPTIMAL CONTROL FOR CANCER DYNAMICS

In this section, two different optimal control approaches, a closed‐loop SDRE and an open‐loop method utilizing steepest descent technique, are applied on the dynamic of cancer in order to figure out which one is the better choice.

### 3.1 Optimal controller design using the closed‐loop SDRE approach

The idea of controller design using SDRE approach is originated from Linear Quadratic Regulator (LQR), in which for a state vector $x\in {ℜ}^{n\times 1}$ and a control signal $u\in {ℜ}^{m\times 1}$ we have,

(2) $$\dot{x}\left(t\right)=A\left(t\right)x\left(t\right)+B\left(t\right)u\left(t\right)$$

where $A\in {ℜ}^{n\times n}$and $B\in {ℜ}^{n\times m}$. This could be used either for linear plant dynamics or for the linearized dynamic of nonlinear systems, which is obtained based on Taylor series expansion of each term [32]. The first step in finding the optimum solution of a system is to define a cost function according to the system's aim in the interval of [*t*_0_−*t*_*f*_] that generally is written in the following form,

(3) $$\begin{array}{l} J=\frac{1}{2}x^{T}\left(t_{f}\right)Hx\left(t_{f}\right)+\\ \frac{1}{2}\int_{t_{0}}^{t_{f}}\left(x{\left(t\right)}^{T}Q\left(t\right)x\left(t\right)+u{\left(t\right)}^{T}R\left(t\right)u\left(t\right)\right)dt \end{array}$$

where *H* and *Q*are real symmetric semi‐definite, and *R* is real symmetric positive weighting matrices for states and control signals. Note that the cost function must be in quadratic form. Generally, weighting matrices are diagonal matrices which *q*_*ii*_ (*r*_*jj*_ or *h*_*kk*_) represents the importance of *x*_*i*_(*t*) (*u*_*jj*_ or *x*_*kk*_(*t*_*f*_)) in the purpose, unless term *x*_*i*_(*t*)*x*_*j*_(*t*) (*u*_*i*_ *u*_*j*_ or *x*_*i*_(*t*_*f*_)*x*_*j*_(*t*_*f*_), i≠j) has a physical meaning and is important in the cost function. The aim is to find **x** ^∗^ and **u** ^∗^such that the cost function is minimised. It is worth noting that the phrase within the integral minimises the states and control signals during simulation and matrix *H* minimises states at final time. According to the studies in Riccati equation, the following relation must be calculated:

(4) $$A^{T}P+PA-PBR^{-1}B^{T}P+Q=0$$

where $P\in {ℜ}^{n\times n}$ is a real symmetric positive definite matrix that is to be calculated. Whenever *P* is obtained, the optimal control vector is determined from Equation (5).

(5) $$u_{opt-LQR}=-R^{-1}B^{T}Px$$

As mentioned earlier, in order to utilize LQR for nonlinear dynamics, the Taylor expansion is used with eliminating nonlinear terms, which in some cases does not provide acceptable answer due to the large approximations. SDRE could be counted as an advanced LQR approach. The advantage of this method is that all nonlinear terms are preserved. The first step is to turn the system dynamics into below form:

(6) $$\dot{x}\left(t\right)=A\left(x\right)x\left(t\right)+B\left(x\right)u\left(t\right)$$

Upon comparing (2) with (6), it is concluded that the elements of *A*(*x*) and *B*(*x*) matrices contain nonlinear terms of ODEs, and their values depends on the states at each time that makes the controller closed‐loop. Then, the following SDRE must be solved to determine matrix *P*(*x*):

(7) $$\begin{array}{l} A^{T}\left(x\right)P\left(x\right)+P\left(x\right)Ax-\\ P\left(x\right)B\left(x\right)R^{-1}\left(x\right)B^{T}\left(x\right)P\left(x\right)+Q\left(x\right)=0 \end{array}$$

where *P*(*x*), *R*(*x*) and *Q*(*x*) have the same properties as mentioned before, but are dependent to states during simulation. Finally, the control signal vector is calculated by (8), which is similar to (5) with the difference of state dependent matrices.

(8) $$u_{opt-SDRE}=-R^{-1}\left(x\right)B^{T}\left(x\right)P\left(x\right)x$$

Remark 1 In nonlinear systems, choosing *A*(*x*) and *B*(*x*)to produce the dynamics into the form of (6) is not unique. The choice must be such that controllability of matrix pairs *A*(*x*) and *B*(*x*) would be undeniable for all x∈Ω. In other words, matrix [*B*(*x*) *A*(*x*)*B*(*x*) ... *A*_*n*−1_(*x*)*B*(*x*)] must be full rank pointwise [33].

Remark 2 In order to calculate matrix *P,* the MATLAB routine “lqr” can be used. The advantage of using this command is that the Riccati equation [see Equation (7)] is calculated by defining *A*,*B*, *Q* and *R* on each time step. Then, $K≜-R^{-1}\left(x\right)B^{T}\left(x\right)P\left(x\right)$ is displayed as the output of the written code.

As discussed above, the first step is to define *A*(*x*) and *B*(*x*) such that it is be controllable. In addition to that, when simulation is running, it is programmed to display an error message if the matrix is not full rank in any time step. By considering $x≜{\left[x_{1}\;x_{2}\;x_{3}\;y\;w\right]}^{\;T}$ as the state vector and $u≜{\left[\delta \;\phi \right]}^{\;T}$ as the control vector, matrix *A*(*x*) is selected as,

(9) $$A\left(x\right)=\left[\begin{array}{lllll} A_{11} & A_{12} & A_{13} & A_{14} & A_{15}\\ A_{21} & A_{22} & A_{23} & A_{24} & A_{25}\\ 0 & \beta & A_{33} & 0 & A_{35}\\ A_{41} & A_{42} & 0 & -\xi & 0\\ 0 & 0 & A_{53} & 0 & -\eta \end{array}\right]$$

where,

(10) $$\begin{array}{l} A_{11}=\alpha_{1}\left(1-x_{1}\right),A_{12}=-q_{1}x_{1},A_{13}=-\frac{p_{11}x_{1}y}{a_{1}+x_{1}},\\ \;A_{14}=-\frac{p_{10}x_{1}}{a_{1}+x_{1}},A_{15}=-\frac{p_{12}x_{1}y}{a_{1}+x_{1}},\\ A_{21}=-q_{2}x_{2},A_{22}=\alpha_{2}\left(1-\frac{x_{2}}{1+\gamma x_{3}}\right),\\ A_{23}=-\frac{p_{21}x_{2}y}{a_{2}+x_{2}},A_{24}=-\frac{p_{20}x_{2}}{a_{2}+x_{2}},\\ A_{25}=-\frac{p_{22}x_{2}y}{a_{2}+x_{2}},A_{33}=\alpha_{3}\left(1-x_{3}\right),\\ A_{35}=-\frac{p_{3}x_{3}}{a_{3}+x_{3}},A_{41}=-\frac{d_{1}y}{a_{1}+x_{1}},\\ A_{42}=-\frac{d_{2}y}{a_{2}+x_{2}},A_{53}=-\frac{d_{3}w}{a_{3}+x_{3}} \end{array}$$

and *B*(*x*) is chosen as,

(11) $$B\left(x\right)=\left[\begin{array}{ll} 0 & 0\\ 0 & 0\\ 0 & 0\\ 1 & 0\\ 0 & 1 \end{array}\right]$$

The last step is to define matrices *Q* and *R*, that are based on system's aim. The main goal is to reduce CCs as quickly as possible, which must be included in matrix *Q*.. It is obvious that if we just consider this term in the cost function, the control signals increase to make the goal happen. As a result, the increment rate of drug delivery would harm the healthy tissue which put the life of patient at risk. Therefore, it is rational to consider minimising control efforts as well by tuning the values of matrix *R*. For more precautions, the elements of matrix *Q* regarding to the states *y* and *w* are also considered to be non‐zero.

The last assumption is that because the period of treatment is specified by doctor before therapy started, the treatment is considered known. With above assumptions, the following matrices are defined for this controller:

(12) $$Q=\left[\begin{array}{lllll} 0 & 0 & 0 & 0 & 0\\ 0 & 1 & 0 & 0 & 0\\ 0 & 0 & 0 & 0 & 0\\ 0 & 0 & 0 & 1 & 0\\ 0 & 0 & 0 & 0 & 10 \end{array}\right],R=\left[\begin{array}{ll} 1 & 0\\ 0 & 5 \end{array}\right]$$

which makes the cost function as:

(13) $$J=\frac{1}{2}\int_{t_{0}}^{t_{f}}\left(x_{2}^{2}+y^{2}+10w^{2}+\delta^{2}+5\phi^{2}\right)dt$$

It should be noted that the optimal control signals vector will be calculated as the product of matrix K and state vector, regarding to Remark 2.

### 3.2 Controller design using calculus of variations and steepest descent method

In this section, another optimal controller basis is considered. The same as previous section, a cost function must be defined depends on the assumptions. In order to utilise calculus of variation to find the optimum solution, a continuous system as (14) is considered,

(14) $$\dot{x}\left(t\right)=a\left(x\left(t\right),u\left(t\right),t\right)$$

The general form of cost function is as follows.

(15) $$J=h\left(x\left(t_{f}\right),t_{f}\right)+\int_{t_{0}}^{t_{f}}g\left(x\left(t\right),u\left(t\right),t\right)dt$$

which is familiar with Equation (3). All the discussion mentioned in Section 3.1 is also valid here. Variables that have a high impact level have grater values and vice versa (The same use as matrix *Q* and *R* in Equation (3)).

To find the extremum of above cost function (*x* ^∗^and the corresponding *u* ^∗^), the Hamiltonian functional for the system is defined as [32].

(16) $$\begin{array}{l} Η\left(x\left(t\right),u\left(t\right),p\left(t\right),t\right)≜\\ g\left(x\left(t\right),u\left(t\right),t\right)+P^{T}a\left(x\left(t\right),u\left(t\right),t\right) \end{array}$$

where *P* = [*P*_1_ *P*_2_ *P*_3_ *P*_4_ *P*_5_]^ *T*^ and *P*_*i*_ s' are co‐state (Lagrangian multipliers). The following equations are derived to be solved for finding the control signal.

- ODEs for system dynamics (state‐space equations, see (1))

(17) $${\dot{x}}^{*}\left(t\right)=\frac{\partial Η}{\partial p}\left(x^{*}\left(t\right),u^{*}\left(t\right),p^{*}\left(t\right),t\right)$$

- ODEs for co‐states

(18) $${\dot{p}}^{*}\left(t\right)=-\frac{\partial Η}{\partial x}\left(x^{*}\left(t\right),u^{*}\left(t\right),p^{*}\left(t\right),t\right)$$

- Algebraic equation for control signals

(19) $$0=\frac{\partial Η}{\partial u}\left(x^{*}\left(t\right),u^{*}\left(t\right),p^{*}\left(t\right),t\right)$$

The remaining point is the boundary conditions (BCs) for above ODEs, which is the key to solving differential equations. For states, the initial conditions are defined based on the system's state when the time started. However, the BCs for co‐states are derived by [32]:

(20) $$\begin{array}{l} {\left[\frac{\partial h}{\partial x}\left(x^{*}\left(t_{f}\right),t_{f}\right)-P^{*}\left(t_{f}\right)\right]}^{T}\delta x_{f}+\\ {}[Η\left(x^{*}\left(t_{f}\right),u^{*}\left(t_{f}\right),P^{*}\left(t_{f}\right),t_{f}\right)+\\ \frac{\partial h}{\partial t}\left(x^{*}\left(t_{f}\right),t_{f}\right)]\delta t_{f}=0 \end{array}$$

In order to apply calculus of variation method on the dynamics of cancer, similar to SDRE, the first step is to define a proper cost function based on the aim of problem. As mentioned earlier, the main goal is to reduce the number of CCs. According to the assumptions stated in Section 3.1 about the limitation of the control inputs amount, and considering the problem as a final time fixed‐final states free one, Hamiltonian could be written as follows,

(22) $$\begin{array}{l} {\dot{P}}_{1}=-P_{1}\left(\alpha_{1}\left(1-x_{1}\right)-\alpha_{1}x_{1}-q_{1}x_{2}-\frac{p_{1}x_{4}}{a_{1}+x_{1}}+\frac{p_{1}x_{1}x_{4}}{{\left(a_{1}+x_{1}\right)}^{2}}\right)+P_{2}q_{2}x_{2}+P_{4}\left(\frac{d_{1}}{a_{1}+x_{1}}-\frac{d_{1}x_{1}}{{\left(a_{1}+x_{1}\right)}^{2}}\right)x_{4}\\ {\dot{P}}_{2}=-2k_{1}x_{2}+P_{1}q_{1}x_{1}-P_{2}(\alpha_{2}\left(1-\frac{x_{2}}{1+\gamma x_{3}}\right)-\frac{\alpha_{2}x_{2}}{1+\gamma x_{3}}-q_{2}x_{1}-\frac{p_{2}x_{4}}{a_{2}+x_{2}}+\frac{p_{2}x_{2}x_{4}}{{\left(a_{2}+x_{2}\right)}^{2}})\\ -P_{3}\beta +P_{4}\left(\frac{d_{2}}{a_{2}+x_{2}}-\frac{d_{2}x_{2}}{{\left(a_{2}+x_{2}\right)}^{2}}\right)x_{4}\\ {\dot{P}}_{3}=-\frac{P_{2}\alpha_{2}x_{2}^{2}\gamma }{{\left(1+\gamma x_{3}\right)}^{2}}-P_{3}\left(\alpha_{3}\left(1-x_{3}\right)-\alpha_{3}x_{3}-\frac{p_{3}x_{5}}{a_{3}+x_{3}}+\frac{p_{3}x_{3}x_{5}}{{\left(a_{3}+x_{3}\right)}^{2}}\right)+P_{5}\left(\frac{d_{3}}{a_{3}+x_{3}}-\frac{d_{3}x_{3}}{{\left(a_{3}+x_{3}\right)}^{2}}\right)x_{5}\\ {\dot{P}}_{4}=\frac{P_{1}p_{1}x_{1}}{a_{1}+x_{1}}+\frac{P_{2}p_{2}x_{2}}{a_{2}+x_{2}}-P_{4}\left(-\xi -\frac{d_{1}x_{1}}{a_{1}+x_{1}}-\frac{d_{2}x_{2}}{a_{2}+x_{2}}\right)\\ {\dot{P}}_{5}=\frac{P_{3}p_{3}x_{3}}{a_{3}+x_{3}}-P_{5}\left(-\eta -\frac{d_{3}x_{3}}{a_{3}+x_{3}}\right) \end{array}$$

(21) $$Η=k_{1}x_{2}^{2}\left(t\right)+k_{2}\delta^{2}+k_{3}\phi^{2}+\sum_{i=1}^{i=5}P_{i}{\dot{x}}_{i}\left(t\right)$$

where *k*_*i*_ is a positive constant which represents the efficacy of its corresponding variable in the Hamiltonian.

The second step is to specify three sets of equations (see Equations (17)–(19)). The ODEs of states are derived based on Equation (17), which is the dynamics of the system [see Equation (1)]. The co‐states' ODEs which are obtained based on Equations (18) and (21) are calculated in Equation (22).

Defining BCs for above ODEs is inseparable in this step. For states, *x*_1_(0) = 0.6, *x*_2_(0) = 0.6, *x*_3_(0) = 0, *y*(0) = 0 and *w*(0) = 0 are chosen as initial conditions based on the study in Reference [29]. Since the treatment is assumed to be a final time fixed‐final states free problem, the co‐states’ BCs are obtained based on Equation (20) as below.

(23) $${\left[\frac{\partial h}{\partial x}\left(x^{*}\left(t_{f}\right),t_{f}\right)-P^{*}\left(t_{f}\right)\right]}^{T}=0$$

Since *h* = 0 according to the assumptions, it is obvious that all co‐states’ values must be zero at final time. The difference of states and co‐states calculations appears here, that states are defined in initial time, but co‐states in final time. This point makes the analytical solution impossible for most problems, especially nonlinear ones. Therefore, numerous numerical methods are used to make sure that all the ODEs along with their BCs and optimal control signals are obtained based on all the facts mentioned above.

As states above, there are five conditions that must be satisfied in calculus of variations optimal problems: The states' and co‐states’ ODEs with their BCs and the algebraic equation for control signals [see Equation (19)]. Each numerical method considers several of these five conditions known, and the remaining is calculated based on the trial and error procedure. At the end of each step, these values are compared with their exact ones and will be updated for the next step. This will continue until the stopping condition is satisfied. In steepest descent method, all ODEs along with their BCs are considered known, and control signals will be corrected by a mathematical basis expressed in Remark 3.

Remark 3 Gradient vector represents the variation of a function, such that the direction of the vector is perpendicular to the function in each point, and its orientation is in the direction of the greatest increment of the function. By knowing the fact that, first, the aim in optimal problems is to find *x* ^∗^ and *u* ^∗^ such that Hamiltonian is minimised (Pontryagin principle) [32].

Therefore, the control signals are updated according to equation (24) with an initial guess for the desired control trajectory.

(24) $$u^{\left(i+1\right)}\left(t\right)=u^{\left(i\right)}\left(t\right)-\tau \frac{\partial Η}{\partial u}\left(t\right)$$

where τ is the rate that specifies the speed of movement. The large value represents the quick steps towards the extremum of the Hamiltonian. Larger steps might result in skipping the minimum value and divergence of the algorithm. Therefore, it is reasonable to choose a large value for the beginnings and a small one for the remaining of the procedure.

According to Hamiltonian [see Equations (21) and (24)], the rate of CA delivery is obtained by

(25) $$\delta^{\left(i+1\right)}\left(t\right)=\delta^{\left(i\right)}\left(t\right)-\tau_{1}\left(2k_{2}\delta^{\left(i\right)}+P_{4}\right)$$

and the rate of AA injection is calculated by

(26) $$\phi^{\left(i+1\right)}\left(t\right)=\phi^{\left(i\right)}\left(t\right)-\tau_{2}\left(2k_{3}\phi^{\left(i\right)}+P_{5}\right)$$

where $\tau_{1}$ and $\tau_{2}$ are the time constants and are chosen 1/(*iteration*)^0.3^ and 1/*iteration* respectively, such that the step number is the denominator of the fractions.

Remark 4 Since the mathematical model of cancer is non‐dimensional, control signals must vary within the range of zero to one. Note that the negative control signal concept is not admissible in cancer models. This constraint should be checked after calculating the control signals from each method. A saturation function is used for the values beyond the limit.

## 4 SIMULATION RESULTS AND DISCUSSION

**FIGURE 1:**
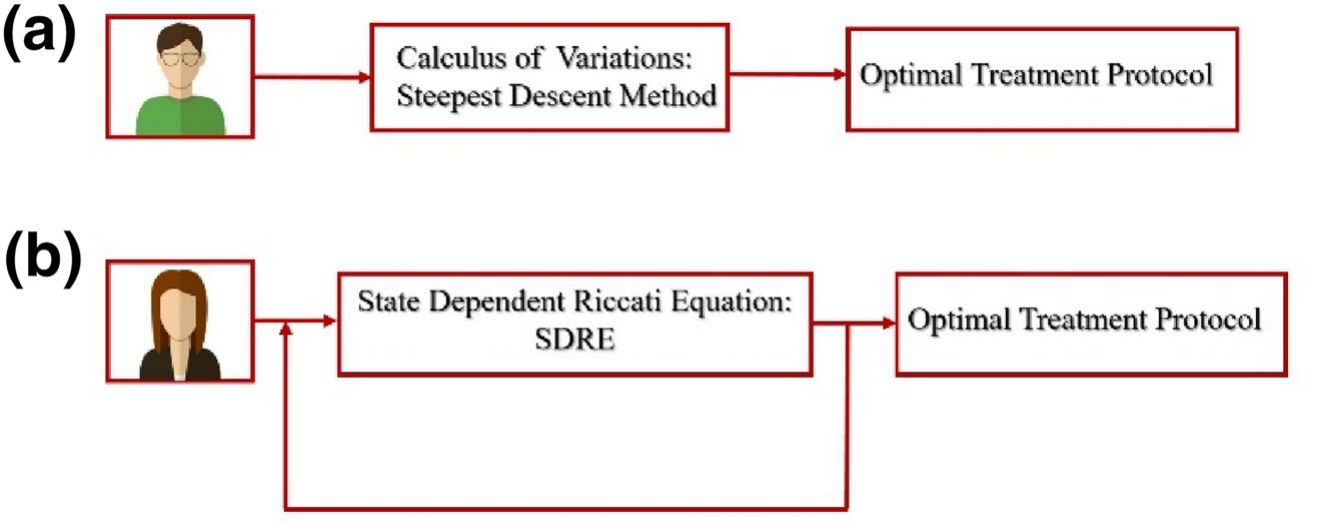
Illustration of the system diagram and the proposed controllers: (a) Calculus of variation method, (b) state‐dependent Riccati equation (SDRE)

In this section, the computer simulation results are discussed in detail. Before talking about the behaviour of the system, let us pay attention to Figure 1. In this figure, the application of the proposed controllers has been illustrated. The aim of the controllers is to identify the optimal treatment protocol. Since calculus of variation method is an open‐loop approach, the controller is just affected by the patient's initial conditions. However, the closed‐loop SDRE has the feedback to overcome the uncertainties.

The SDRE matrices are defined in Section 3.1 and the calculus of variation approach and steepest descent method are in Section 3.2, respectively. For steepest descent method, the control signals are updated based on Equations (25) and (26) with 400 steps of iteration.*k*_1_ = 0.043, *k*_2_ = 0.5 and *k*_3_ = 0.001 are selected for Equation (21) from trial and error method. The control signals are validated in 250 days of treatment for both controllers.

**FIGURE 2:**
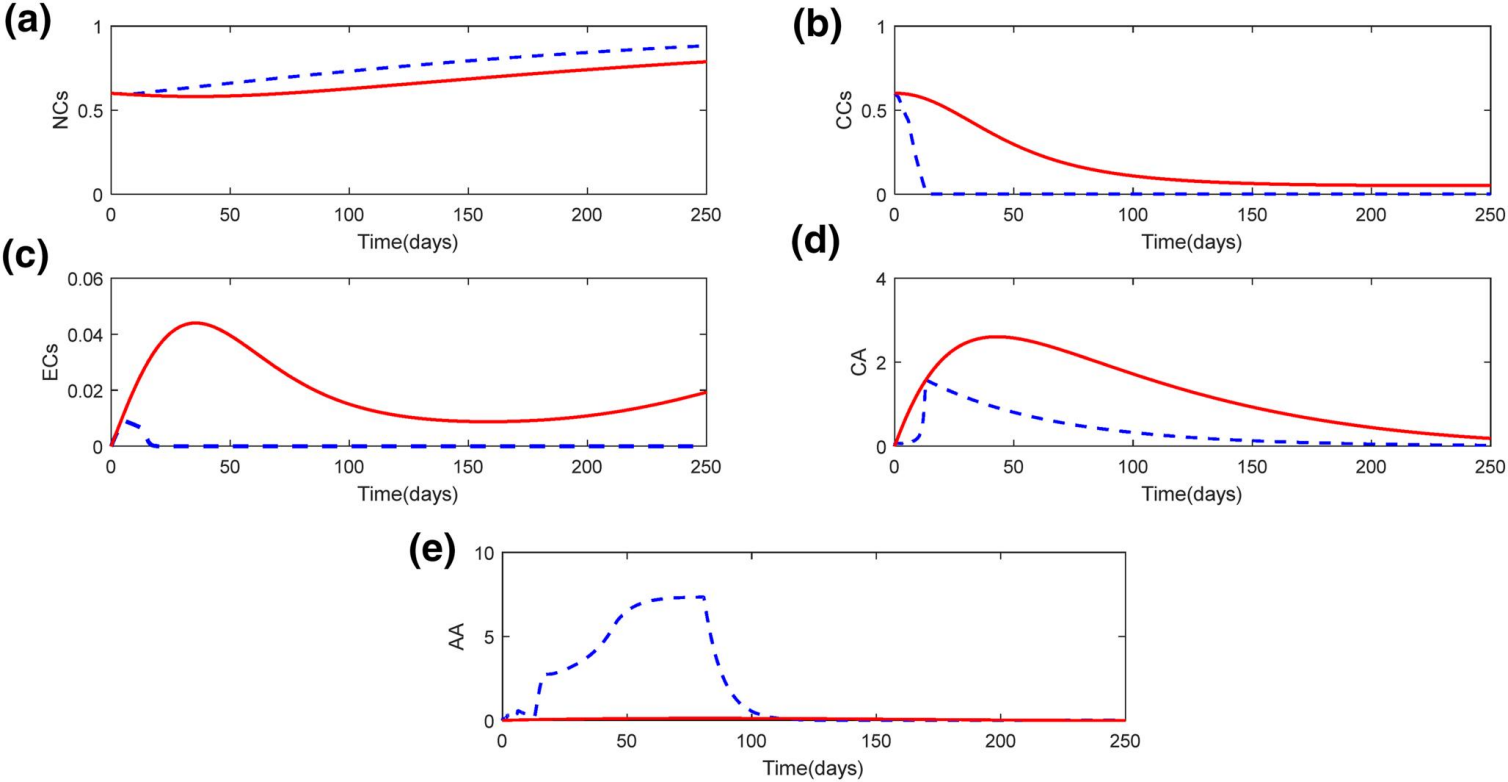
State variation in cancer treatment for state‐dependent Riccati Equation (SDRE) (dashed‐line) and steepest descent method (thick‐line) approaches with initial conditions *x*_1_(0) = 0.6, *x*_2_(0) = 0.6, *x*_3_(0) = *y*(0) = *w*(0) = 0: (a) normal cells, (b) cancer cells, (c) endothelial cells, (d) chemotherapy agent, (e) anti‐angiogenic agent

Figure 2 illustrates all the states in SDRE (dashed‐line) and steepest descent method (thick‐line). The variation of each state is compatible with the mathematical model behaviour in both optimal controllers. In both curves, NCs have a downward trend in the beginning of treatment (Figure 2a). This is because of the CCs invasion to healthy tissues of body. Since CCs improvement in body is controlled (Figure 2b), NCs experienced an increment trend in both methods and tends to its desire value, one; its non‐dimensional quantity based on the model assumptions in Section 2. However, a difference between the values of the two curves in a specific time is visible. Since in SDRE method, CCs has reached zero earlier, and the corresponding NCs converge to one. It should be noted that if the treatment duration is increased in steepest descent method, the concentration of NCs would be closer to one.

CC s’ variation results with time in both controllers are shown in Figure 2b. Descending trend of CCs is one of the criterion that indicates the both optimal control goals have been achieved. However, the final value in steepest descent method did not reach zero. It is also valid, since All the effects or other simultaneous treatments, like radiotherapy or immunotherapy, are not considered in the mathematical model. In real life experience, if the tumour still remains after therapies, it is extracted from the body by a surgeon. The key point in this part is that optimal control signals are found such that are capable of stopping CCs proliferation in patient's body.

In steepest descent method, ECs has three different behaviours with the passage of time: First, it has an increasing trend, which is because of the existence of CCs in body due to angiogenesis. Second, a decreasing trend which is the effect of AA against CCs in body. This indicates that ECs are controlled by AA, although CCs make ECs to branch out. As AA vanishes in body (Figure 2e), the third behaviour of ECs appear. As a result, ECs start growing. Hence, the ECs' proliferation at this stage is not caused by CCs, because CCs have a constant amount at this time (Figure 2b).

Even though, ECs variation is a little bit different for SDRE controller. The third behaviour in steepest descent method is not visible here (Figure 2c). After the system is controlled, ECs remain zero until the end of the treatment, which is caused by the different amount of AA in body in the two controlled methods (Figure 2e). Since the amount of AA in body is greater for SDRE, its corresponding ECs has a lower value, and eventually reaches zero. As mentioned in Section 2, AA is the only drug effective on ECs. Therefore, the increment in the amount of AA results in decreasing the value of ECs quickly. The reason for the differences of AA for both control methods are due to the difference in their amounts of control signals.

As shown in Figure 2d, CA has a similar behaviour in both control methods. The increment of CA in the first part is the main reason of CCs decrement. The descending part of the curves is due to the steady state of the system, which is mainly focused on CCs values (Figure 2b). As stated in Section 2, CA is the key drug which destroys tumour in body and is an aid to patient's immune system, and, this is completely clear in all curves in Figure 2 and explanations above.

As mentioned earlier, the system is controlled by two control signals, injection rate of CA (δ) and AA (ϕ) at a specific time. Both are calculated by means of optimal controllers, as stated in Section 3. It is shown that in the beginning of treatment, both controllers produce a nonzero value in order to eliminate CCs from body. And eventually reach zero, since CCs behaviour are steady.

**FIGURE 3:**
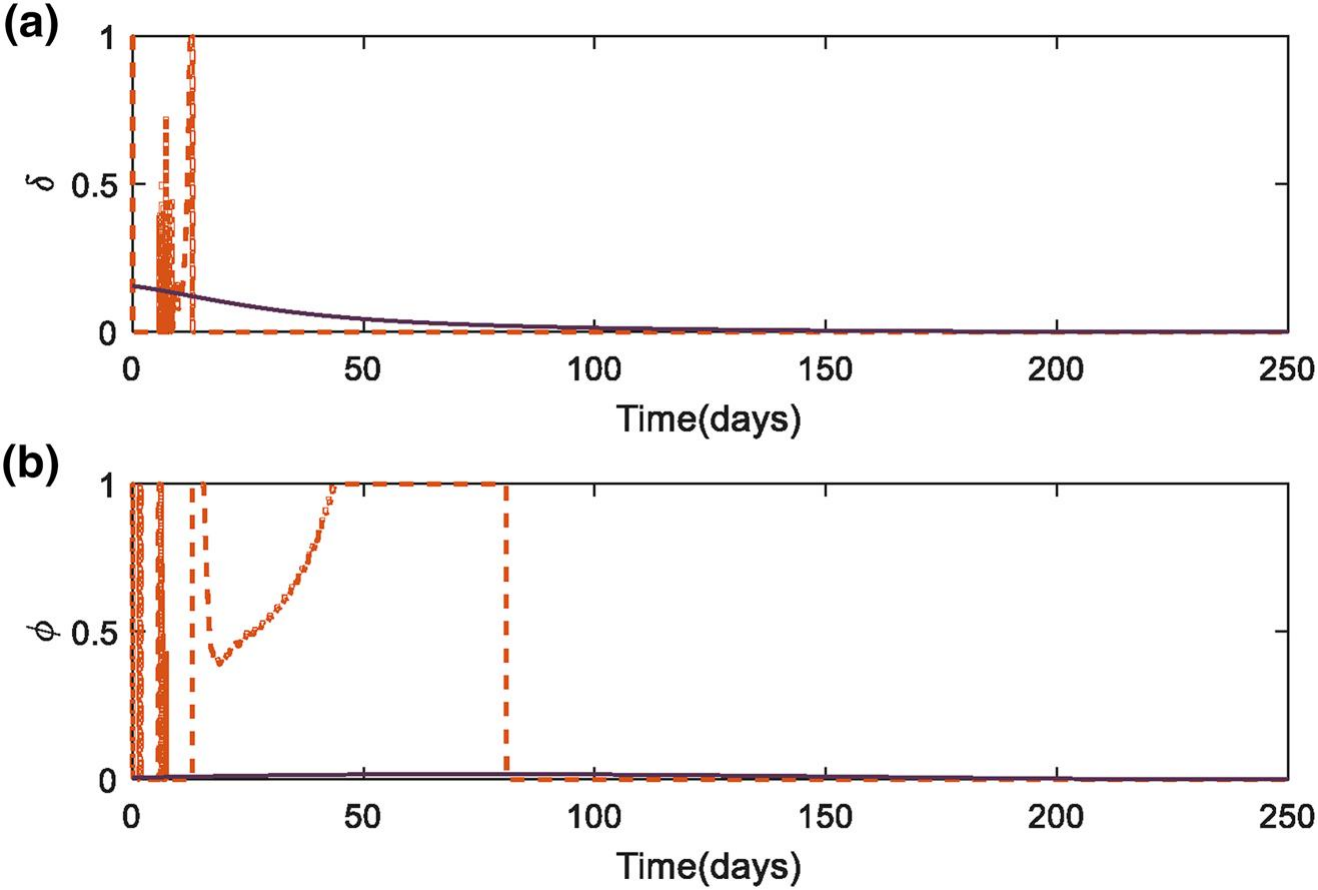
Control signal variation in cancer treatment for state‐dependent Riccati equation (SDRE) (dashed‐line) and steepest descent method (thick‐line): (a) Injection rate of chemotherapy agent (CA), (b) Injection rate of anti‐angiogenic agent (AA)

By considering the points stated in Section 2 about the amounts of injected drugs, it could be realized that the amount of CA in body must be greater than AA because of the main responsibility it has. This is also proved by experimental data in [34]. Thus, by comparing curves on Figures 3a and 3b for each method, it can be concluded that steepest descent method has a better performance. It should be mentioned that the matrices Qand *R*are ways to minimize the peak of their corresponding state or signals.

Another consideration is to check whether continuous signals in treatment are valid or not. It may be a bit far from reality that these two control signals have been considered continuous throughout this time. This assumption has been widely used in many other articles to examine and compare control methods and different models. According to [35], the first theoretical research that assumed continuous drug delivery in cancer chemotherapy is [36], which found it reliable.

It's worth‐noting that the saturation for control signals mentioned in Remark 4, is just used in SDRE controller. The signals obtained from steepest descent method are in the range of zero to one, which is due to proper initial guesses (Figure 3).

**FIGURE 4:**
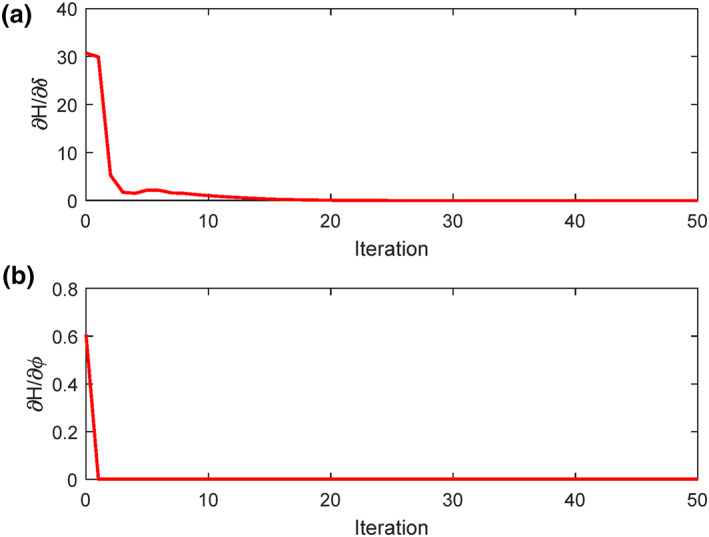
Verification of stopping condition based on step number of calculations in steepest descent method: (a) ∂Η/∂δ, (b) ∂Η/∂ϕ

The optimal solution (states and control signals) in both methods are biologically justified. In steepest descent method another assessment must be considered in order to realize whether the solution calculated is mathematically converged or not. As mentioned in Section 3.2, it was noted that a stopping condition must be considered. The value that can be used as the evaluation of the answer is $\partial Η/\partial u_{i}$, where *u*_*i*_ is a control signal. Thus, two values of ∂Η/∂δ and ∂Η/∂ϕ are also calculated and plotted in Figures 4a and 4b, respectively. As shown in this figure, these two values reach zero at the initial time steps (until 50 iterations are plotted), which indicates the rapid convergence of the answer to this problem. Therefore, the total solution of steepest descent method is mathematically converged and biologically acceptable.

As stated earlier, the SDRE advantage is that the control inputs calculated are closed‐loop. In other words, the controller will find the next optimal solution based on states' variations on previous time step. This is not true for Steepest Descent method. Since control signals are calculated from the co‐states’ values (see (25) and (26)) and co‐states are varied with time, this method is open‐loop. In order to verify this point for the proposed SDRE in this paper, the following disturbances assumed for the CCs:

(27) $$d\left(x_{2},t\right)=\left\{ \begin{array}{ll} 0 & 0\leq t\leq 100\\ 0.001 & 100\leq t\leq 150\\ 0.009 & 150\leq t\leq 200\\ 0 & 200\leq t\leq 400 \end{array}\right.$$

**FIGURE 5:**
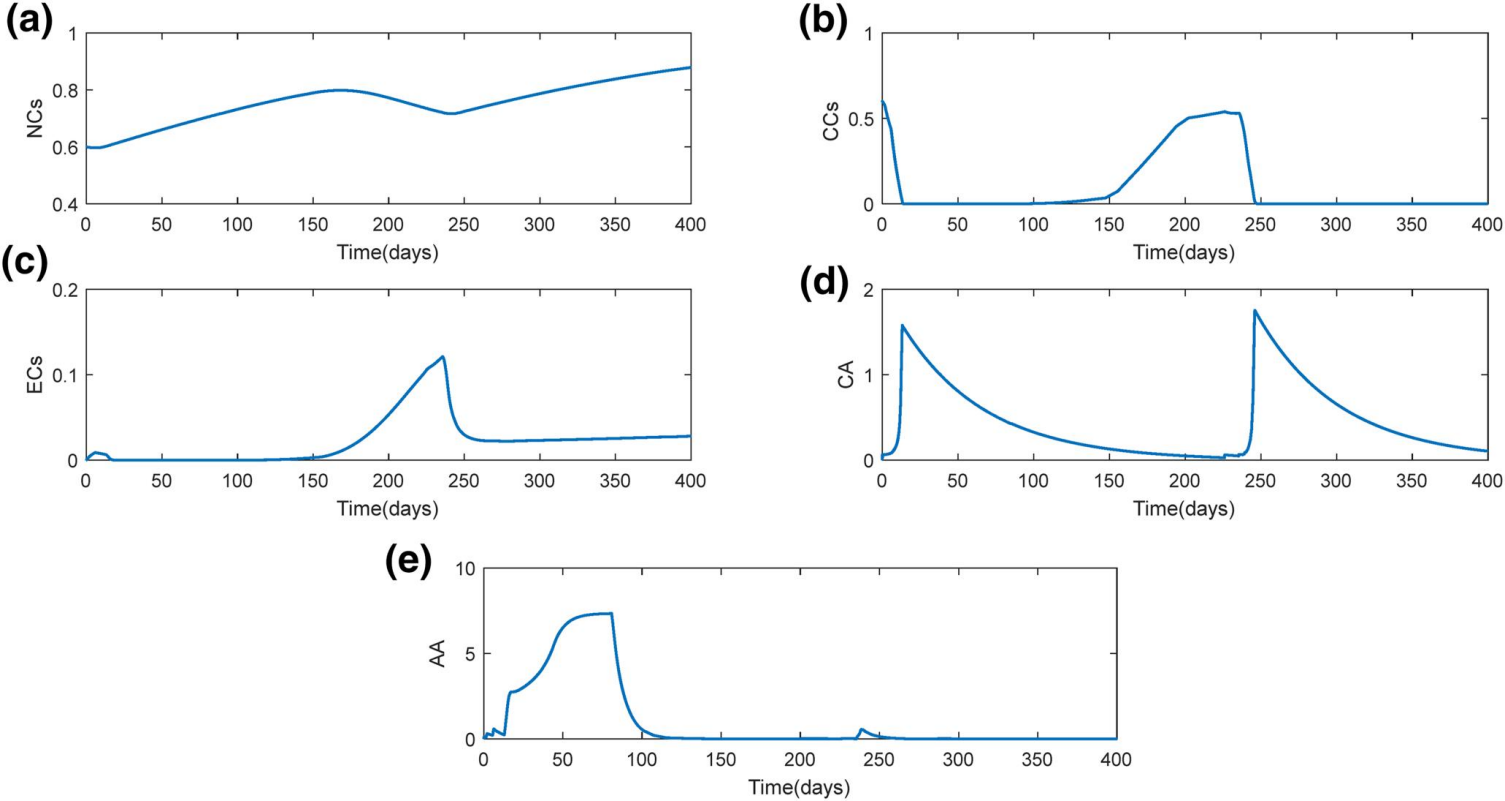
State variation for disturbed cancer system using disturbance distribution with time in Reference (27) utilising SDRE approach with initial conditions: *x*_1_(0) = 0.6, *x*_2_(0) = 0.6, *x*_3_(0) = *y*(0) = *w*(0) = 0*:* (a) Normal cells, (b) Cancer cells, (c) Endothelial cells, (d) Chemotherapy agent, (e) Anti‐angiogenic agent

The disturbance is assumed to be on the critical state (CCs), in case the tumour resists against therapies, and has an ascending trend from the day 100 to 200. The controller calculates the optimum signals from the same matrices for undisturbed system and the states behaviour is illustrated in Figure 5.

As the disturbance is applied, ECs have ascending trend (Figure 5c) which cause CCs to increase (Figure 5b), that shows the condition of patient is worse. As a result, NCs decrease (Figure 5a), which puts the life of patient in risk. The descending behaviour of NCs is due to the increment of CCs and CA in body. CA is injected to destroy CCs, but as it does not distinguish between CCs and NCs. CA must be injected to control the tumour growth (Figure 5d). The amounts of AA in body increase in order to destroy ECs that are reproduced (Figure 5e). The total response of the system appears before day 250^th^. After this day, CCs decrease and other states, except NCs, converge to the desired value.

**FIGURE 6:**
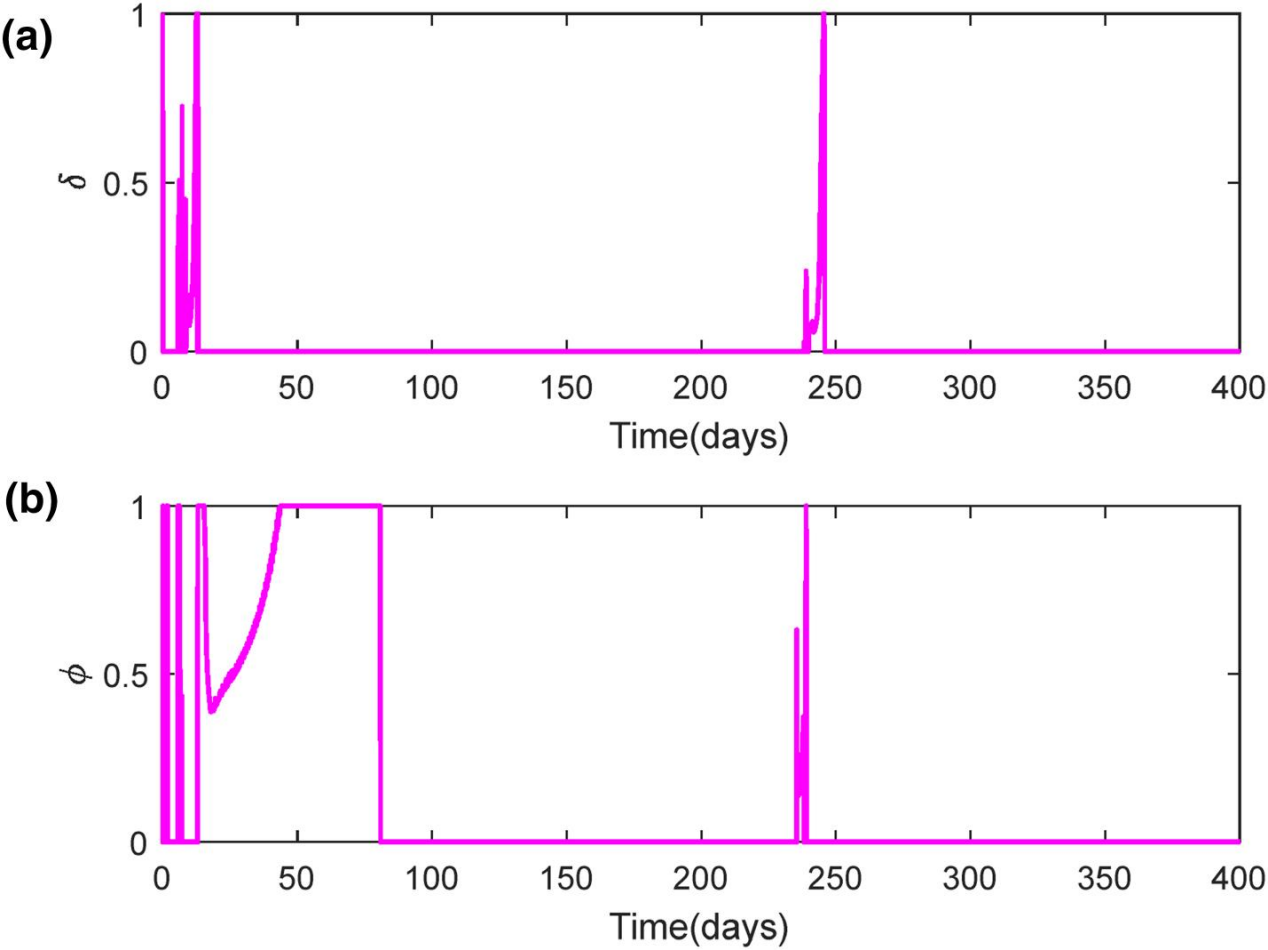
Control signals for disturbed cancer system: (a) Injection rate of CA, (b) Injection rate of AA

The variation of control signals obtained by SDRE method are illustrated in Figure 6. The behaviour is similar to Figure 3 in initial days. However, the disturbed part is visible, since the signals' values are non‐zero within this time. The result of these signals is satisfactory since the aim is completely achieved.

Remark 5 It should be noted that SDRE controller can only eliminate small disturbances, and if the amount of disturbances increases suddenly and the tumour becomes very large, SDRE cannot provide an adequate optimal rate of drug injection for the treatment of the disease. In such condition, other therapies are suggested to enhance the performance of chemotherapy and anti‐angiogenic.

### 4.1 Comparison with previous works

There are numerous works in literature that study cancer mathematical models in order to investigate the behaviour of their states with optimal approach. The aim is unanimous: Diagnosing the optimal treatment protocols such that the body's immune system with the aid of injected drugs would be capable of reducing CCs into the safe amount and also minimising the usage of drugs at the end of the therapy sessions. There are several constraints that must be satisfied during the therapy: NCs which is indicative of the patient's health must be kept at a reasonable level the whole time. All the state variables and control signals must be positive during the treatment, since the negative state would be meaningless. These points along with the complexities of the mathematical models, challenges identifying the optimal protocol with satisfying the mentioned goals and constraints. In this section, we review similar works in this field.

A state space model (a four‐population model: tumour cells, host (normal) cells, immune cells, and drug interaction) was presented and analysed in References [13,37] by De Pillis and co‐workers, and the optimal control therapy behaviour of the system was compared with the traditional pulsed periodic treatment. The effect of using the SDRE controller on the De Pillis's mathematical model and using different weight matrices was investigated in Reference [38], and the same controller was used in a Model Reference Adaptive Controller (MRAC) to adapt the system to the desired path in order to overcome the uncertainties [39]. The effect of obesity was then considered in Reference [10] which is mostly based on De Pillis's model, and then an optimal approach was applied to the system by defining a cost function and constructing Hamiltonian in calculus of variation method, in order to investigate the effects of low and high caloric diet on chemotherapy treatment protocols [14]. This optimum trajectory was then used in Reference [15] in order to build a desired sliding surface.

De Pillis's state space model had one control signal with four or five (fat cells) states to control. It' is obvious with complexity of the dynamic system and the mentioned constraints, and it' is a very difficult task. Therefore, the Pinho's model (a five‐population model: tumour cells, NCs, ECs, chemotherapy and anti‐angiogenic agents) which was presented in [29] with two control signals (the rate of chemotherapy and anti‐angiogenic injection) is considered. This model was considered in Reference [30] in order to find the adaptive control signals with the existence of uncertainties, which guide the system towards the optimal trajectory.

To sum up, identifying the optimal control approach which leads to find the optimal treatment protocol is a very important task not only in presenting a reliable rule‐based chemotherapy protocol but also in constructing the foundation in MRAC or for estimating system dynamic parameters based on an optimal desired path, which has its own benefits. That is why the two optimal approaches were considered and compared in this paper to validate the pros and cons of each. The aim is to highlight the points which can be very useful in choosing the suitable optimal controller not only in cancer dynamics but also in every system dynamic.

## 5 CONCLUSIONS AND FUTURE WORKS

In this study, a new cancer dynamic in the presence of chemotherapy and anti‐angiogenic effects is considered for cancerous tumour growth control. For the first time, the effects of two mentioned treatments are seen, and then, two optimal approaches are designed for the considered mathematical model in order to reduce the amount of CCs as the main control aim of this study. The selected optimal control methods are basic optimal control method based on calculus of variation along with steepest descent method as an open‐loop control strategy and SDRE as a closed‐loop one. The basis of each method is described based on the needs, and each optimal control signal is designed based on a proper cost function for cancer dynamics. Afterwards, behaviour of states and system's response analysis were explained in details for each control method in simulation section.

The validation of the solutions of the optimal controllers' results was assessed biologically and mathematically and found acceptable for both. It was observed that although the basic optimal controller is a powerful method and has a mathematical background, however since it is an open‐loop controller, if disturbances happen, the system cannot respond properly. On the other hand, although the background of SDRE is not as strong as calculus of variation in finding the optimal solution, but it is capable of handling the disturbances and uncertainties to the system. It is worth‐noting that in SDRE approach on the point‐wise controllability and stability of the closed‐loop system is a very complicated issue to verify because the matrices of the system are varying with time and with control inputs every step time. Another point which makes this method hard to apply is that because the dynamic system is nonlinear, the matrices are not unique and can be chosen differently with too many possibilities, each of them would definitely affects the controllability, stability of the closed‐loop system, and of course, the amount of control signals. Thus, it is a long journey to find the system matrices which satisfy the controllability and stability of the close‐loop system in every time step. Whereas, calculus of variation approach is independent of the system linearity or nonlinearity, and its implementation is rather easy and straightforward.

As pointed in Remark 4, the control signals must have a saturation function to make sure that the control signals are in an acceptable range. Figure 2 shows that SDRE has used the saturation function which undoubtedly would have effects on the controllability and stability of the system. Although it is clear in Figure 3 that the open‐loop controller did not use the saturation function, it is worth‐noting that if the control signals are not in the defined range, we will use the constrained problem in calculus of variations [32], which has its own story.

Thus, in one hand, calculus of variation has a strong mathematical background with a straightforward method, which is flexible in constrained problems and easy to apply, yet it is open‐loop. On the other hand, SDRE approach is closed‐loop and able to overcome possible uncertainties, whereas applying it is rather difficult, since it is challenging to specify the system matrices, checking the controllability and stability of the system in every times step, and not flexible enough in constrained problems.

Therefore, from above comments and the necessity of choosing the right optimal approach (mentioned in Section 4.1), it can be concluded that if an optimal controller could be designed in order to mix the advantage of both methods, the solution can be widely used in finding the optimum injection drug rates and predict the behaviour of system dynamics in the field of cancer treatment. For example, if the calculus of variation approach is applied in every time step, in order to make sure the possible uncertainties are taking into account at the moment, the final response will be more reliable. However, if the final response exceeds the limits, constrained calculus of variation must be used instead.

In future works, an observer can be added to the system in order to estimate the states that are unmeasurable. As mentioned above, laboratory data can be added in order to classify the patient's conditions and investigate the efficiency and performance of the results of the closed‐loop control system for various situations.

### 5.1 Limitations of the study

It should be noted that this work can be the beginning of a wide work for personalizing the treatment protocol in chemotherapy which is optimum for each patient. The optimal controller must be flexible enough to handle the probable disturbances (which is very common in cancerous patients) during chemotherapy sessions. In order to achieve this goal, enough laboratory data on mice and then humans are needed to improve the proposed protocol. This paper could be the start of this idea with the hope of helping these patients more quickly with a specific optimum goal than the current protocol. It is worth noting that since the conventional learning algorithms do not have any mathematical model‐based background, it would not give us any variation of states with respect to time. The advantage of considering the mathematic model and utilising it to drive the optimum control signal is the availability of calculating the variations of ther unmeasurable states (ECs e.g.) during treatment sessions, which could be counted as a validation of the final answer. As mentioned above, the unmeasurable states could be estimated by means of an observer and laboratory data in order to get closer to reality, which is in the schedule of the upcoming study.
